# Supplementary material for: Co-infection of Dengue, Zika and Chikungunya in a group of pregnant women from Tuxtla Gutiérrez, Chiapas: Preliminary data. 2019
Source: PLoS Negl Trop Dis. 2020 Dec 21;14(12):e0008880. doi: 10.1371/journal.pntd.0008880 (PMC7785221; doi:10.1371/journal.pntd.0008880)
Supplement: Attachment — Submitted filename: Response.pdf [file pntd.0008880.s001.pdf]

Hospital Infantil de México Federico Gómez
Instituto Nacional de Salud
Laboratorio de Investigación en Parasitología

June 26th, 2020

Dear Editor
PLOS Neglected Tropical Diseases

I inform you that the manuscript No. PNTD-D-20-00205 Co-infection of Dengue, Zika and Chikungunya in a group of pregnant women from Tuxtla Gutiérrez, Chiapas: preliminary data. 2019. All the pregnant women included in the study were explained what the objective of the investigation was and were asked to sign a letter of informed consent for the use of the samples. They all signed regardless of age.

I hope to successfully meet the requeriments.

Best regards

Dr. Enedina Jiménez Cardoso PhD

Dr. Márquez 162, Col. Doctores, 06720, Cuauhtémoc, Ciudad de México
55 5228 9917 ext. 2375 y 2376 www.himfg.edu.mx

2020 AÑO DE LEONA VICARIO BENEMÉRITA MADRE DE LA PATRIA
