# Supplementary material for: Co-infection of Dengue, Zika and Chikungunya in a group of pregnant women from Tuxtla Gutiérrez, Chiapas: Preliminary data. 2019
Source: PLoS Negl Trop Dis. 2020 Dec 21;14(12):e0008880. doi: 10.1371/journal.pntd.0008880 (PMC7785221; doi:10.1371/journal.pntd.0008880)
Supplement: Attachment — Submitted filename: Response to requeriments.pdf [file pntd.0008880.s003.pdf]

Hospital Infantil de México Federico Gómez
Instituto Nacional de Salud
Laboratorio de Investigación en Parasitología

September 29th, 2020

Dear Editor
PLOS Neglected Tropical Diseases

I send you the responses to requirements in the manuscript No. PNTD-D-20-00205R2 Co-infection of Dengue, Zika and Chikungunya in a group of pregnant women from Tuxtla Gutiérrez, Chiapas: preliminary data. 2019:

1. **Figure Files:**

Please upload your figure files to the Preflight Analysis and Conversion Engine (PACE) digital diagnostic tool, https://pacev2.apexcovantage.com. PACE helps ensure that figures meet PLOS requirements.

R= We have upload the figure files to the preflight Analysis and conversion engine (PACE) digital diagnostic tool and later the figures were attached in the resubmission system

2. **Data Requirements:**

Please note that, as a condition of publication, PLOS' data policy requires that you make available all data used to draw the conclusions outlined in your manuscript. Data must be deposited in an appropriate repository, included within the body of the manuscript, or uploaded as supporting information. This includes all numerical values that were used to generate graphs, histograms etc.

R= We have included as an attachment the information data, that includes all numerical values that were used to generate graphs, tables and figures.

3. **Reproducibility:**

To enhance the reproducibility of your results, PLOS recommends that you deposit laboratory protocols in protocols.io, where a protocol can be assigned its own identifier (DOI) such that it can be cited independently in the future.

R= The protocols of the research work have been deposited in protocols.io and the DOI number that was generated is: dx.doi.org/10.17504/protocols.io.bmvhk636

I hope to successfully meet the requirements.

Dr. Enedina Jiménez Cardoso PhD
