# Supplementary material for: Co-infection of Dengue, Zika and Chikungunya in a group of pregnant women from Tuxtla Gutiérrez, Chiapas: Preliminary data. 2019
Source: PLoS Negl Trop Dis. 2020 Dec 21;14(12):e0008880. doi: 10.1371/journal.pntd.0008880 (PMC7785221; doi:10.1371/journal.pntd.0008880)
Supplement: Attachment — Submitted filename: ResponsestoPlossept.docx [file pntd.0008880.s002.docx]

**Responses to requirements**

**Manuscript ID: PNTD-D-20-00205R2**

1. **We ask that a manuscript source file is provided at Revision. Please upload your manuscript file as a .doc, .docx, .rtf or .tex. If you are providing a .tex file, please upload it under the item type ‘LaTeX Source File’ and leave your .pdf version as the item type ‘Manuscript’.**

**R=** The manuscript is uploaded as .doc and we also included it in a .pdf version.

2. **Please provide all main figures in .tif or .eps format in the File Inventory. Please remove the embedded figures from your manuscript. For more information about how to convert your figure files please see our guidelines: http://journals.plos.org/plosntd/s/figures. Once you've converted your files to .tif or .eps, please also make sure that your figures meet our format requirements:** [**http://journals.plos.org/plosntd/s/figures#loc-file-requirements**](http://journals.plos.org/plosntd/s/figures#loc-file-requirements)**.**

**R=** All figures was uploaded in .tif format as individual files. The requirements for sending figures were reviewed and adapted to send them properly. The figures embedded in manuscript were removed and included in separately files as requested. Captions were included in manuscript.

3. **We do not publish any copyright or trademark symbols that usually accompany proprietary names, eg (R), (C), or TM (e.g. next to drug or reagent names). Therefore please remove all instances of trademark/copyright symbols throughout the text, including TM on page 9.**

**R=** All instances of trademark/copyright symbols throughout the text, including TM on page 9 were removed.

4. **Thank you for including an Ethics Statement for your study. Please include a statement that formal consent was obtained from the parent/guardian (must state whether verbal/written) OR the reason consent was not obtained (e.g. anonymity).**

**R=** The statement “Written consent and assent was obtained from all participants, which was signed either by themselves or a responsible family member” was included in lines 159-161 in methodology section.
